# Understanding Landmarking and Its Relation with Time-Dependent Cox Regression

**DOI:** 10.1007/s12561-016-9157-9

**Published:** 2016-07-11

**Authors:** Hein Putter, Hans C. van Houwelingen

**Affiliations:** Department of Medical Statistics and Bioinformatics, Leiden University Medical Center, PO Box 9600, 2300 Leiden, RC The Netherlands

**Keywords:** Landmarking, Time-dependent covariates, Time-dependent Cox regression

## Abstract

Time-dependent Cox regression and landmarking are the two most commonly used approaches for the analysis of time-dependent covariates in time-to-event data. The estimated effect of the time-dependent covariate in a landmarking analysis is based on the value of the time-dependent covariate at the landmark time point, after which the time-dependent covariate may change value. In this note we derive expressions for the (time-varying) regression coefficient of the time-dependent covariate in the landmark analysis, in terms of the regression coefficient and baseline hazard of the time-dependent Cox regression. These relations are illustrated using simulation studies and using the Stanford heart transplant data.

## Introduction

Time-dependent covariates play an important role in the analysis of censored time-to-event data. Prominent examples include the effect of heart transplant on survival for heart patients [6] and the effect of CD4+ T-cell counts on the occurrence of AIDS or death for HIV-infected patients [15]. In the first example the time-dependent covariate is a binary covariate, in the second example a numerical covariate, typically measured longitudinally. These two examples constitute the most common instances of time-dependent covariates in survival analysis. Examples in the context of organ transplantation include the aforementioned heart transplant example, but also the effect of kidney transplantation or changing dialysis modality in end-stage renal disease, or the effect of graft failure on survival for patients with a liver transplant.

Broadly speaking, two approaches have come in general use in estimating the effect of time-dependent covariates. The first is time-dependent Cox regression, already mentioned by [5]. In this approach the hazard at time *t* is assumed to depend on the current value at time *t* of the time-dependent covariate, *X*(*t*), through the product of a baseline hazard and $\exp (\beta X(t))$. This approach yields valid inference if the value of the time-dependent covariate is known for all subjects at all event time points without error, and the regression model is correctly specified. Especially in the longitudinal setting, this is typically not the case and many researchers have studied the amount of bias when measurement error and ageing of covariates are present [2, 11, 15]. A second approach is landmarking [3], which involves setting a landmark time point *s*, and using the value of the time-dependent covariate at *s* (or using some other appropriate summary of the history of the time-dependent covariate up to *s*) as a time-fixed covariate in an analysis of survival from *s* onwards, in a subset of subjects at risk at *s*. For overviews we refer to [7, 12].

In principle, both approaches can be used for estimating the effects of time-dependent covariates, and there are no clear settings where the choice between time-dependent Cox and landmarking is obvious. Each of the two methods has its advantages and disadvantages. To get some feeling of the relative merits of the two approaches, let us consider the situation of the Stanford heart transplant example [6]. Patients are admitted to a waiting list, the time to event is time from admittance to the waiting list until death, which may be subject to censoring, and interest is in the effect of a heart transplant on survival. The time-dependent covariate heart transplant, *X*(*t*), is thus initially equal to 0, and attains the value 1 as soon as the patient receives a heart transplant. (If the patient never receives a heart transplant, the value remains 0 throughout his/her follow-up.) The most important reason for the popularity of landmarking, especially in the present context of a binary time-dependent covariate, is its transparency. It is clear what is being compared: at the landmark time *s*, two groups are compared with regard to their survival from time *s* onwards, one group without, the other group with a heart transplant received before or at time *s*. Differences between these two groups can be visualized by plotting the Kaplan–Meier survival curves for the two groups. In contrast, such a visualization is much less obvious for the time-dependent Cox model. Survival curves can only be shown for patients with $X(t) \equiv 0$ and $X(t) \equiv 1$, respectively. Model-free curves have also been proposed in this context, sometimes referred to as Simon–Makuch curves [13]. Both model-based and Simon–Makuch curves show the survival for fictional patients who either never receive a heart transplant ($X(t) \equiv 0$) or have received a heart transplant at $t=0$ ($X(t) \equiv 1$). In both cases one may question whether these curves reflect clinically realistic quantities. The landmarking curves have a much clearer interpretation; see also [4] for a detailed discussion on this topic. Disadvantages of landmarking are first of all the need for a (to some extent arbitrary) choice of landmark time point, and also a loss of power because, especially for later landmark time points, subjects with an event before the landmark time point are excluded from analysis. In many cases, early landmark time points also lead to a loss of power, because in the beginning of follow-up, there will be few subjects with a heart transplant, leading to highly unbalanced groups. For estimation of the effect of a heart transplant on survival, both methods are equally applicable. The topic of this paper is the following: suppose that a time-dependent Cox regression model is valid. In a landmark analysis at landmark time *s* two groups, one with ($X(s)=1$) and one without ($X(s)=0$) a heart transplant are compared. Those subjects without a heart transplant at time *s* might in the future receive one, so that $X(t)=1$ for some future *t*, whereas for subjects with a heart transplant at time *s*, *X*(*t*) will keep the value 1. This will make the groups with $X(s)=0$ and $X(s)=1$ more similar in the future and will therefore attenuate the effect of the time-dependent covariate, compared to the time-dependent Cox regression.

The purpose of this paper is to understand, quantify and illustrate the differences between the regression coefficients of a time-dependent Cox model and those obtained in a landmark analysis. Starting from a time-dependent Cox regression model, which we assume to be correctly specified, we will derive formulas for the (time-varying) regression coefficient corresponding to the landmark model. We study two special cases of time-dependent covariates in more detail and show that if the time-dependent Cox model satisfies the proportional hazards assumption, there will be attenuation in the sense that the landmark regression coefficient is between the time-dependent Cox regression coefficient and 0. We show that the degree of attenuation depends on the rate of change of the time-dependent covariate. An illustration using the Stanford heart transplant data is provided.

## Theory

Let *T* denote the time-to-event random variable of interest. Let *X*(*t*) denote a time-dependent covariate, which for simplicity we take to be one-dimensional, and denote its complete history until time *t* by $\overline{X}(t) = \{ X(s); 0 \le s \le t\}$. We assume that the hazard of *T*, conditional on $\overline{X}(t)$, is given by

$$\begin{aligned} h(t \,|\,\overline{X}(t)) = \lim _{\mathrm{\;d}t\downarrow 0} \mathrm{P}(T \le t + \mathrm{\;d}t\,|\,T \ge t)/\mathrm{\;d}t= h_0(t) \exp (\beta (t) X(t)), \end{aligned}$$

i.e. the hazard at time *t* is assumed to depend on the whole history $\overline{X}(t)$ only through the present value, and it follows a Cox model with possibly time-varying effect given by $\beta (t)$. The relations to be derived in this paper are valid irrespective of censoring, but for consistent estimation of the parameters in the model and survival predictions it is assumed that censoring is independent of *T* and $X(\cdot )$, possibly given other time-fixed covariates in the model, which have been omitted here for the sake of simplicity.

Fix a time point *s*. A landmark analysis at time *s* will fix the value of $X(\cdot )$ at *X*(*s*), and assume a model for *T* with *X*(*s*) as time-fixed covariate, based on the survivors at risk at time *s* [3]. Independent censoring, and, if appropriate, truncation, is assumed, which implies that the survivors at risk at time *s* are representative for survivors at time *s*. As a result the hazard considered for the landmark model at landmark time *s* is given by

$$\begin{aligned} h_{\mathrm{LM}}\left( t \,|\,s, X(s)\right) = \lim _{\mathrm{\;d}t\downarrow 0} \mathrm{P}(T \le t + \mathrm{\;d}t\,|\,T \ge t, X(s), T \ge s)/\mathrm{\;d}t, \end{aligned}$$

where the additional conditioning on $T \ge s$ is in fact superfluous. It is only retained in the notation of $h_{\mathrm{LM}}(t \,|\,s, X(s))$ to emphasize that the landmark analysis is based on survivors at *s*. The postulated model for this landmark hazard is typically taken to be a proportional hazards model as well:

1 $$\begin{aligned} h_{\mathrm{LM}}(t \,|\,s, X(s)) = h_{\mathrm{LM},0}(t \,|\,s) \exp \big (\beta _{\mathrm{LM}}(t \,|\,s) X(s)\big ). \end{aligned}$$

Often $\beta _{\mathrm{LM}}(t \,|\,s)$ is taken to be time-fixed in the analysis, i.e. $\beta _{\mathrm{LM}}(t \,|\,s) \equiv \beta _{\mathrm{LM}}(s)$, but it may (and typically will) depend on *s*.

The question addressed in this note is as follows: what is the relation between $\beta _{\mathrm{LM}}(t \,|\,s)$ and $\beta (t)$, and how does it depend on $h_0(t)$ and on the development of *X*(*t*)? Intuitively, the landmark model employs an old value, *X*(*s*), instead of the current value *X*(*t*) to describe the hazard at time *t*. As a result, if *X*(*t*) changes rapidly between *X*(*s*) and *X*(*t*) and if *X*(*t*) is strongly related to *T*, one may expect a large discrepancy between $\beta (t)$ and $\beta _{\mathrm{LM}}(t \,|\,s)$. The aim is to quantify how quickly $\beta _{\mathrm{LM}}(t \,|\,s)$ changes for $t \ge s$, depending on the rate of change of *X*(*t*), on $\beta (t)$ and on $h_0(t)$.

Our development will start by considering the conditional survival function at time $t > s$, given survival until time *s* and given *X*(*s*),

$$\begin{aligned} S(t \,|\,s, X(s))= & {} \mathrm{P}(T \ge t \,|\,T \ge s, X(s)) \\= & {} \mathrm{E}\left[ \exp \Bigl ( - \int _s^t h_0(u) e^{\beta (u) X(u)} \mathrm{\;d}u\Bigr ) \, \Bigl | \, X(s) \right] . \end{aligned}$$

The landmark hazard $h_{\mathrm{LM}}(t \,|\,s, X(s))$ is the derivative with respect to *t* of the negative logarithm of $S(t \,|\,s, X(s))$ at $t=s+w$,

2 $$\begin{aligned}&h_{\mathrm{LM}}(t \,|\,s, X(s))\nonumber \\&\quad = \frac{ - \frac{\mathrm{\;d}}{\mathrm{\;d}t} S(t \,|\,s, X(s))}{S(t \,|\,s, X(s))} \nonumber \\&\quad = \frac{\mathrm{E}\left[ h_0(t) e^{\beta (t) X(t)} \,|\,T \ge t, X(s) \right] \cdot \mathrm{E}\left[ e^{- \int _s^t h_0(u) e^{\beta (u) X(u)} \mathrm{\;d}u} \,|\,T \ge s, X(s) \right] }{S(t \,|\,s, X(s))} \nonumber \\&\quad = h_0(t) \mathrm{E}\left[ e^{\beta (t) X(t)} \,|\,T \ge t, X(s) \right] , \end{aligned}$$

provided that, conditional on $T \ge t$ and *X*(*t*), *T* is independent of *X*(*s*). This expression can also be found in [15].

It is possible to further evaluate $h_{\mathrm{LM}}(t \,|\,s, X(s))$ for a number of specific models for the development of *X*(*t*). In what follows we consider two of such models. The first of these is the common situation where *X*(*t*) is dichotomous, the other is a specific case where *X*(*t*) is continuous.

### Dichotomous Time-Dependent Covariates

Let *X*(*t*) be a dichotomous time-dependent covariate. This is the type of situation for which the landmarking approach was originally proposed [3]. In that paper the endpoint was overall survival, and interest was in the effect of response to chemotherapy (a time-dependent covariate, coded as 0 $=$ no response, 1 $=$ response). Many more examples can be given, including the effect of disease recurrence on survival [16], the effect of treatment adherence on disease recurrence [8] or the effect of adverse events on recurrence rates [9].

#### Theory

In this context our aim is to obtain an expression of

3 $$\begin{aligned} h_{\mathrm{LM}}(t \,|\,s, X(s)=g) = h_0(t) \mathrm{E}\left[ e^{\beta (t) X(t)} \,|\,T \ge t, X(s)=g \right] ,\quad \ g = 0,1. \end{aligned}$$

**Fig. 1:**
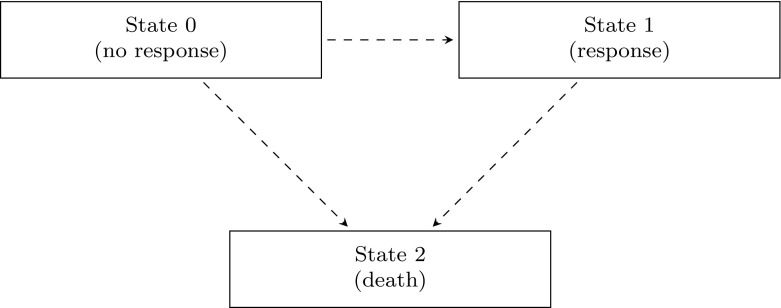
An irreversible illness-death multi-state model, with response as the illness state

The conditional expectation can be evaluated by considering the multi-state model given in Fig. 1.

States 0 and 1 correspond to the values of the time-dependent covariate (response) being 0 and 1, respectively, and state 2 is the death state. The original landmarking paper did not consider a possible transition back from state 1 to 0, but we will allow this here. The relation between the event time *T* and the multi-state model *X*(*t*) is given by the equivalence $\{ X(t) = 2 \} \Leftrightarrow \{ T \le t \}$.

In the present context, Eq. (3) is valid if for those alive at time *t*, conditional on the current state *X*(*t*), the transition to death is independent of *X*(*s*), the state at time *s*. This assumption is fulfilled if the multi-state model is Markov. If that is the case, then the transition probabilities $P_\mathrm{gh}(s,t) = \mathrm{P}(X(t)=h \,|\,X(s)=g)$ can be calculated if the hazards of making a transition from 0 to 1 or backwards and the transitions from 0 or 1 to state 2 (death) are known, using the Kolmogorov–Chapman forward equations. The transition hazard from *g* to *h* will be denoted by $\lambda _\mathrm{gh}(t)$. The conditional *prevalence* probabilities

$$\begin{aligned} \pi _{g1}(s,t)= & {} \mathrm{P}(X(t)=1 \,|\,X(s)=g, T \ge t) \\= & {} \mathrm{P}(X(t)=1 \,|\,X(s)=g, X(t)=0/1) = \frac{P_{g1}(s,t)}{P_{g0}(s,t)+P_{g1}(s,t)}, \end{aligned}$$

defined for $g=0,1$, describe the conditional probability of being in response (in state 1) at time *t*, given in state *g* at the earlier landmark time *s*, and given alive at time *t*. Finally, we define $\pi _{g0}(s,t) = 1 - \pi _{g1}(s,t)$. The conditional expectation in (3) can then be written as

$$\begin{aligned} \pi _{g0}(s,t) + e^{\beta (t)} \pi _{g1}(s,t). \end{aligned}$$

This implies that the hazard ratio in the landmark model can be expressed as

4 $$\begin{aligned} \exp \bigl ( \beta _{\mathrm{LM}}(t \,|\,s) \bigr ) = \frac{h_{\mathrm{LM}}(t \,|\,s, X(s)=1)}{h_{\mathrm{LM}}(t \,|\,s, X(s)=0)} = \frac{\pi _{10}(s,t) + e^{\beta (t)} \pi _{11}(s,t)}{\pi _{00}(s,t) + e^{\beta (t)} \pi _{01}(s,t)}. \end{aligned}$$

A number of remarks can be made regarding this expression. First, if $\beta (t) \equiv 0$, that is, when the time-dependent covariate has no effect at all on survival, then we have $\beta _{\mathrm{LM}}(t \,|\,s) \equiv 0$, independently of $\pi _{g0}(s,t)$ and $\pi _{g1}(s,t)$. Second, the landmark regression coefficient $\beta _{\mathrm{LM}}(t \,|\,s)$ is always between $-\beta (t)$ and $\beta (t)$. A further simplification can be made when considering the most common situation, the irreversible case, where the $1 \rightarrow 0$ transition is not possible, and hence $\pi _{11}(s,t) \equiv 1$. This gives

5 $$\begin{aligned} \exp (\beta _{\mathrm{LM}}(t \,|\,s)) = \frac{e^{\beta (t)}}{\pi _{00}(s,t) + e^{\beta (t)} \pi _{01}(s,t)} = \frac{e^{\beta (t)}}{1 + (e^{\beta (t)}-1) \pi _{01}(s,t)}, \end{aligned}$$

and has $0 \le \beta _{\mathrm{LM}}(t \,|\,s) \le \beta (t)$. The intuitive explanation of the formula is as follows: if those with $X(s)=0$ quickly jump to 1 (so if $\pi _{01}(s,t)$ is high), then the effect is quickly attenuated, so $\beta _{\mathrm{LM}}(t \,|\,s)$ is close to 0, while if those with $X(s)=0$ remain in 0, then the effect is not attenuated, so $\beta _{\mathrm{LM}}(t \,|\,s)$ will be close to $\beta (t)$. The derivative with respect to *t* of $\beta _{\mathrm{LM}}(t \,|\,s)$, evaluated at the landmark *s*, is given by

$$\begin{aligned} \beta ^\prime (s) - \left( e^{\beta (s)} - 1 \right) \cdot \left\{ \lambda _{01}(s) + \lambda _{10}(s) e^{-\beta (s)} \right\} . \end{aligned}$$

If the time-dependent Cox model is correct and satisfies the proportional hazards assumption, i.e. if $\beta (t) \equiv \beta$, then $\beta _{\mathrm{LM}}(t \,|\,s)$ will usually vary over *t*, unless for instance $\beta =0$. As mentioned earlier, usually the effect of the time-dependent covariate is estimated through a proportional hazards model like (1), where the proportional hazards assumption would ignore the possibly time-varying nature of $\beta _{\mathrm{LM}}(t \,|\,s)$. Equations (4) and (5) show that a proportional hazards landmark model would typically be misspecified. If such a misspecified Cox regression landmark model is fitted, then [17–19] the estimate obtained in that model will be approximately equal to

6 $$\begin{aligned} \beta _{\mathrm{LM}}^*(s) \approx \frac{\int _s^{t_\mathrm{hor}}\beta _{\mathrm{LM}}(t \,|\,s) \mathrm{var}(X(t) | T=t) h(t) S(t) C(t) \mathrm{\;d}t}{\int _s^{t_\mathrm{hor}}\mathrm{var}(X(t) | T=t) h(t) S(t) C(t) \mathrm{\;d}t}. \end{aligned}$$

#### Illustration

**Fig. 2:**
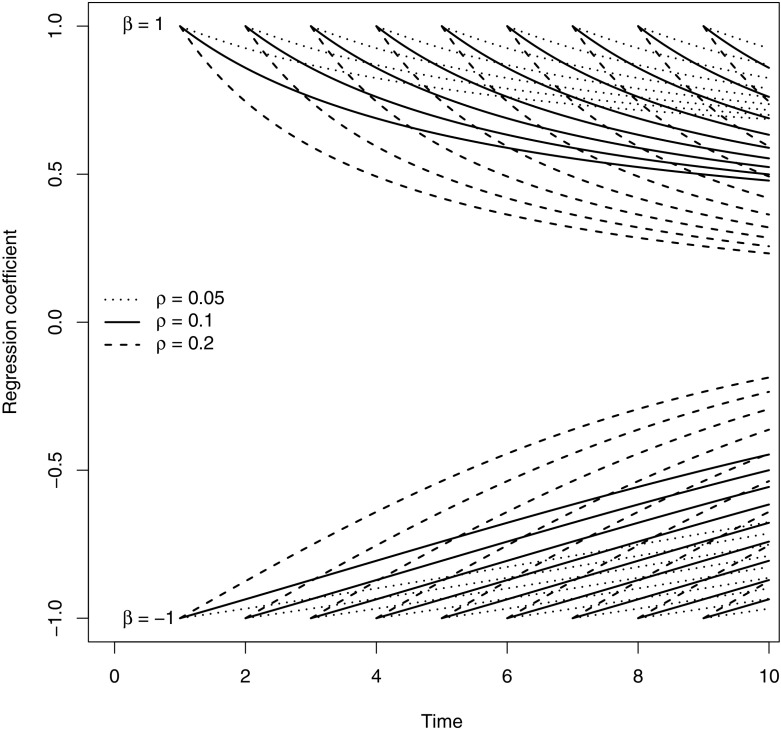
The (time-dependent) landmark regression coefficients (on the *y*-axis) for different values of $\rho$ and $\beta$

Figure 2 shows a plot of $\beta _{\mathrm{LM}}(t \,|\,s)$ of Eq. (5) on the *y*-axis in the irreversible case, where both time to response and time to death follow exponential distributions with different values of the rates $\lambda _{01}(t) \equiv \rho$ for response, and $\beta$; the death rate without response was set to $\lambda _{02}(t) \equiv \lambda = 0.1$. The landmark regression coefficients $\beta _{\mathrm{LM}}(t \,|\,s)$ have been recalculated for different values of *s*.

The attenuation as time between *s* and *t* increases can be clearly seen. The degree of attenuation increases with larger values of $\rho$.

**Fig. 3:**
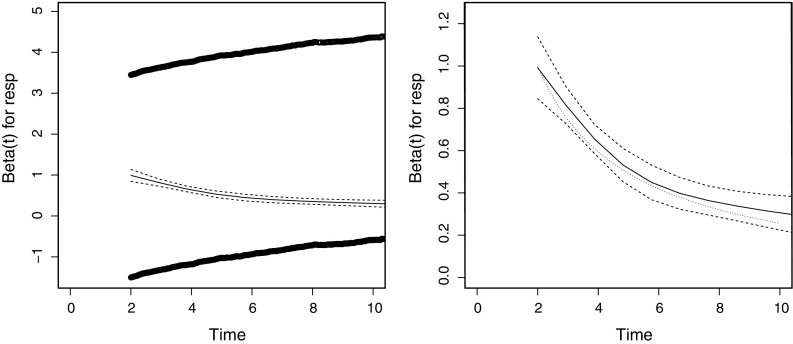
The (time-dependent) landmark regression coefficients from cox.zph

The time-dependent effect of $\beta _{\mathrm{LM}}(t \,|\,s)$ is further illustrated by generating a single large dataset ($n=10,000$) based on the same exponential distributions as before with $\rho =0.2$, $\lambda =0.1$, $\beta =1$. A landmark analysis was performed at $s=2$, with death as endpoint and *X*(*s*), response at 2 years, as time-fixed covariate. Subsequently the method of [10] based on Schoenfeld residuals, as implemented in cox.zph in the survival package [14] in R was applied. This method gives an approximation of $\hat{\beta }_{\mathrm{LM}}(t \,|\,s)$ as function of *t*. Figure 3 shows the result, on the left the plot including the residuals, on the right only the (default) lowess curve in black showing the approximation of $\hat{\beta }_{\mathrm{LM}}(t \,|\,s)$, with 95 % confidence intervals in black dashed lines. In dotted lines the theoretical formula of (4) is shown.

The conclusion is that the theoretical time-varying effect of $\beta _{\mathrm{LM}}(t \,|\,s)$ can be detected and retrieved (the lowess curve being close to the theoretical curve) in a large dataset.

**Fig. 4:**
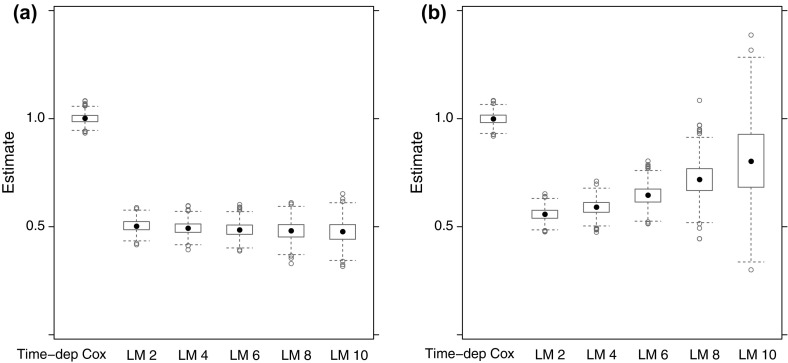
Estimates of $\beta _{\mathrm{LM}}^*(s)$ for $\rho =0.2$, $\lambda =0.1$, $\beta =1$; without (**a**) and with (**b**) censoring

After focusing on the time-varying nature of $\beta _{\mathrm{LM}}(t \,|\,s)$ of the landmark model, we now turn to what is being estimated when (as is usually done in practice) a proportional hazards landmark model is fitted to the data. Figure 4 shows box-plots of the estimates of $\beta _{\mathrm{LM}}^*(s)$ of (6), obtained from 1000 simulations from datasets of 10,000 subjects, with $\rho =0.2$, $\lambda =0.1$, $\beta =1$, for values of $s = 2,4,6,8,10$, together with the time-dependent Cox regression estimate. In Fig. 4a no censoring was applied, while in Fig. 4b independent uniform censoring between 7.5 and 12.5 was applied.

The mean of the estimates of $\beta _{\mathrm{LM}}^*(s)$ hardly changed with *s* (a minimal decrease from 0.504 at $s=2$ to 0.476 at $s=10$). The mean for the time-dependent Cox regression was 1.001. With censoring, the mean of the estimates of $\beta _{\mathrm{LM}}^*(s)$ increased from 0.558 at $s=2$ to 0.808 at $s=10$. The reason for this increase compared to the case of no censoring is that for later landmark times, contributions from $\beta _{\mathrm{LM}}(t \,|\,s)$ for larger *t* get less weight (because of censoring, *C*(*t*) is smaller). With censoring, the mean for the time-dependent Cox regression was 0.999.

**Fig. 5:**
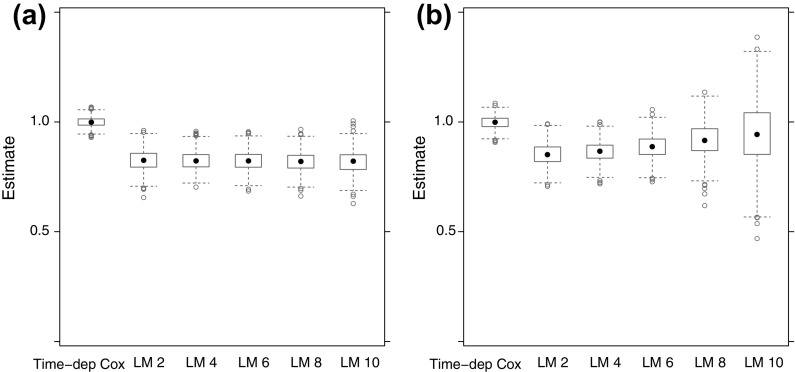
Estimates of $\beta _{\mathrm{LM}}^*(s)$ for $\rho =0.05$, $\lambda =0.1$, $\beta =1$; without (**a**) and with (**b**) censoring

Figure 5 is similar to Fig. 4, the only difference being that now $\rho =0.05$. The difference between the time-dependent Cox and the landmark analyses is now much smaller, because with smaller $\rho$, $\pi _{01}(s,t)$ is now smaller. Without censoring, the mean of the estimates of $\beta _{\mathrm{LM}}^*(s)$ decreased from 0.826 at $s=2$ to 0.819 at $s=10$. With censoring, the means increased from 0.852 at $s=2$ to 0.946 at $s=10$. The means of the time-dependent Cox estimates were 1.000 (no censoring) and 0.998 (censoring).

### Continuous Time-Dependent Covariates

#### Theory

Explicit calculations as for the case of dichotomous time-dependent covariates are much harder to obtain for continuous time-dependent covariates. Recall from  (2)

7 $$\begin{aligned} h_{\mathrm{LM}}(t \,|\,X(s)) = h_0(t) \cdot \mathrm{E}\left( e^{\beta (t) X(t)} \,|\,T \ge t, X(s) \right) . \end{aligned}$$

We will follow [2, 15] in assuming that conditioning on $T \ge t$ is negligible [11] (see also), and that the joint distribution of *X*(*s*) and *X*(*t*) is Gaussian. The results given below should thus be understood as approximations that should be reasonable in low-risk situations. We will derive such approximations for the case where the time-dependent covariate *X*(*t*) follows a Gaussian process with mean $\mu (t) = \mathrm{E}X(t)$ and covariance function $K(s,t) = \mathrm{cov}(X(s),X(t))$. Then the conditional distribution of *X*(*t*) given *X*(*s*) is normal with mean $\mu (t \,|\,X(s)) = \mu (t) + K(s,t) K^{-1}(s,s) (X(s)-\mu (s))$ and variance $\sigma ^2(t \,|\,X(s)) = K(t,t) - K^2(s,t) K^{-1}(s,s)$. The conditional expectation on the right-hand side of (7) is in fact the moment generating function of the conditional distribution of *X*(*t*), given *X*(*s*), evaluated at $\beta (t)$ (recalling that the additional condition that $T \ge t$ is ignored). This leads to the approximation

8 $$\begin{aligned} \mathrm{E}\left( e^{\beta (t) X(t)} \,|\,T \ge t, X(s) \right) \approx \exp \left\{ \beta (t) \mu (t \,|\,X(s)) + \frac{1}{2} \beta ^2(t) \sigma ^2(t \,|\,X(s)) \right\} . \end{aligned}$$

Note again that the approximation in (8) is due to having ignored the selection on having survived to time *t*.

A quite useful special case, also considered in [2], is the case where the time-dependent covariate of subject *i* at time *t* follows

9 $$\begin{aligned} X_i(t) = \mu (t) + b_i + X_i^*(t), \end{aligned}$$

with the mean $\mu (t)$ a deterministic time trend, $b_i$ a random person effect, following a zero mean normal distribution with variance $\omega ^2$, and $X_i^*(t)$ a mean zero Ornstein–Uhlenbeck (OU) process, starting at $X_i^*(0)=0$, and defined further by

10 $$\begin{aligned} \mathrm{\;d}X_i^*(t) = - \theta X_i^*(t) \mathrm{\;d}t+ \sigma \mathrm{\;d}W_i(t), \end{aligned}$$

where $W_i(t)$ is a Wiener process and $\theta$ and $\sigma$ are parameters describing the degree of mean reversal (to zero) and influence of the random fluctuations of the Wiener process, respectively. The solution of (10) is given by [1] (see, e.g.) [A.4]

$$\begin{aligned} X_i^*(t) = \sigma \int _0^t \exp (-\theta (t-s)) \mathrm{\;d}W_i(s). \end{aligned}$$

This is a stationary Wiener process with covariances

$$\begin{aligned} \mathrm{cov}(X_i^*(s),X_i^*(t)) = \frac{\sigma ^2}{2\theta } \exp (-\theta |t-s|). \end{aligned}$$

Adding the random person effect *b*, the result is a stationary Wiener process with

$$\begin{aligned} \mathrm{cov}(X_i(s),X_i(t)) = \omega ^2 + \frac{\sigma ^2}{2\theta } \exp (-\theta |t-s|) = \sigma ^2_{\mathrm{tot}} \left( \rho + (1-\rho )\exp (-\theta |t-s|)\right) , \end{aligned}$$

where $\sigma _{\mathrm{tot}}^2 = \omega ^2 + \sigma ^2/(2\theta )$ is the total variance of *X*(*t*) and $\rho = \omega ^2 / \sigma _{\mathrm{tot}}^2$ is the intraclass correlation, the proportion of the total variance represented by the random person effect variance. The correlation

11 $$\begin{aligned} \rho (s,t) = \rho + (1-\rho )\exp (-\theta |t-s|) \end{aligned}$$

drives the conditional distribution of $X_i(t)$, given $X_i(s)$, which can be seen to be normal with mean $\mu (t \,|\,X_i(s))$ and variance $\sigma ^2(t \,|\,X_i(s))$, given by

$$\begin{aligned} \mu (t \,|\,X(s))= & {} \mu (t) + \rho (s,t) \left( X(s) - \mu (s) \right) ,\\ \sigma ^2(t \,|\,X(s))= & {} \sigma _{\mathrm{tot}}^2 \left( 1-\rho ^2(s,t) \right) . \end{aligned}$$

Using (8), this leads to the approximation

12 $$\begin{aligned}&\mathrm{E}\left( e^{\beta (t) X(t)} \,|\,T \ge t, X(s) \right) \nonumber \\&\approx \exp \biggl [ \beta (t) \left\{ \mu (t) + \rho (s,t) ( X(s) - \mu (s) ) \right\} + \frac{1}{2} \beta ^2(t) \sigma _{\mathrm{tot}}^2 \left\{ 1-\rho ^2(s,t) \right\} \biggr ]. \end{aligned}$$

Taking logarithms in (12) and differentiating with respect to *X*(*s*) yields

13 $$\begin{aligned} \beta _{\mathrm{LM}}(t \,|\,s) = \frac{\mathrm{\;d}}{\mathrm{\;d} X(s)} \ln h_{\mathrm{LM}}(t \,|\,X(s)) \approx \beta (t) \rho (s,t). \end{aligned}$$

So, when the time-dependent covariate follows (9)–(10), the regression coefficient $\beta _{\mathrm{LM}}(t \,|\,s)$ in the landmark model is approximately equal to the regression coefficient $\beta (t)$ in the time-dependent Cox model, attenuated by the correlation function $\rho (s,t)$, defined in (11). The correlation function $\rho (s,t)$, defined in (11), decreases exponentially from 1 to $\rho$ as *t* increases from *s* to $\infty$, the speed of decay depending on $\theta$.

#### Illustration

**Fig. 6:**
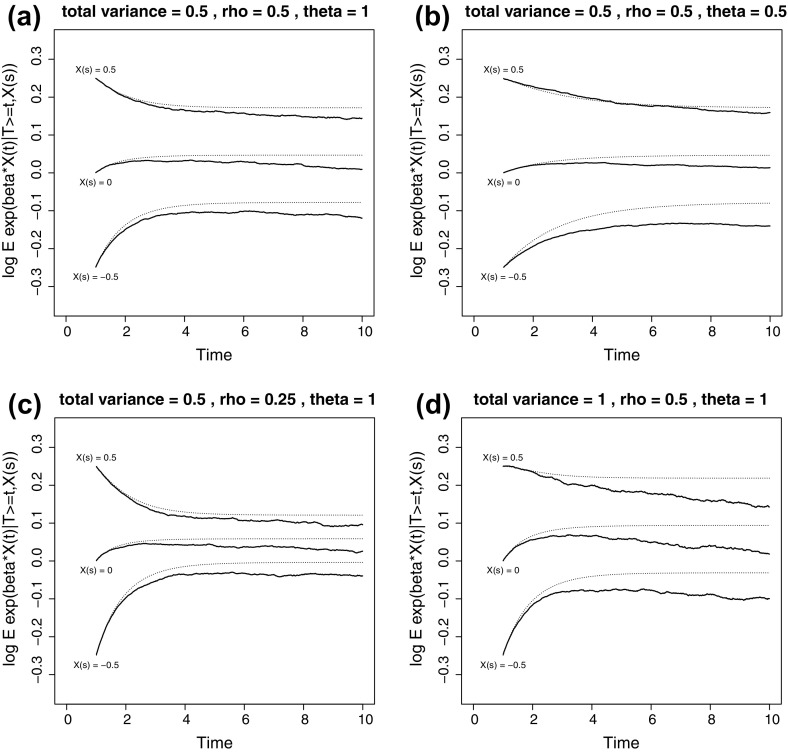
Theoretical and Monte Carlo approximations of $\mathrm{E}\left( e^{\beta (t) X(t)} \,|\,T \ge t, X(s) \right)$ for reference setting [**a** $\sigma ^2_{\mathrm{tot}} = 0.5$, $\rho = 0.5$, $\theta = 1$], and for different choices of $\theta =1$ (**b**), $\rho = 0.25$ (**c**), and $\sigma ^2_{\mathrm{tot}} = 1$

Four plots in Fig. 6 illustrate the approximation in (12) for a relatively simple situation with one time-dependent covariate. The baseline hazard $h_0(t)$ was taken to be constant, equal to 0.1, corresponding to an exponential distribution with mean 10. The hazard at time *t*, given the history $\overline{X}(t)$, was taken to be

14 $$\begin{aligned} h(t \,|\,\overline{X}(t)) = h_0(t) \exp (\beta X(t)), \end{aligned}$$

with $\beta = 0.5$. Figure 6a shows the reference situation where the trajectories of the time-dependent covariate were generated according to an OU process, with $\mu (t) \equiv 0$, total variance $\sigma _{\mathrm{tot}}^2 = 0.5$, intraclass correlation $\rho = 0.5$ and $\theta =1$. For given $\sigma _{\mathrm{tot}}^2$, $\rho$ and $\theta$, the random person effect variance $\omega ^2$ was chosen such that $\omega ^2 / \sigma _{\mathrm{tot}}^2 = \rho$, and $\sigma ^2$ such that $\omega ^2 + \sigma ^2/(2\theta ) = \sigma _{\mathrm{tot}}^2$. The landmark was fixed at $s=1$. The approximation in (12) is contrasted with a Monte Carlo approximation where an OU process with the parameters specified above was generated, jointly with an event time following (14). For each of a very large number of subjects, $i=1,\ldots ,N$, first a random effect $b_i$ was generated according to a mean zero normal distribution with variance $\omega ^2$. The starting value of the realization of $X^*_i(t)$ was set to $X^*_i(0) = 0$. Subsequently, for a series of very short intervals of length $\mathrm{\;d}t$ (we took 0.01), given the current value of $X^*_i(t)$, the value of $X_i(t)$ was set at $b_i + X^*_i(t)$, the hazard was calculated according to (14), and a coin was tossed with probability $h_0(t) \exp (\beta X_i(t)) \mathrm{\;d}t$ to decide if the subject would die in that interval. If not, a new value for $X^*_i(t + \mathrm{\;d}t)$ was calculated according to $X^*_i(t) - \theta X^*_i(t) \mathrm{\;d}t+ \sigma U \mathrm{\;d}t$, with *U* an independent standard normal random variable, and $X_i(t + \mathrm{\;d}t)$ was set as $b_i + X^*_i(t + \mathrm{\;d}t)$. The procedure for subject *i* stopped when the subject died or at a censoring time of 10 years. A very large database of processes and associated death or censoring times was stored. For a given landmark time point *s*, here 1 year, the number of evaluable subjects (i.e. surviving until *s*) was set at 500,000. Conditioning on $X(s) = 0$ was achieved by considering only those simulated processes for which *X*(*s*) was less than $\mathrm{\;d}X$ away from 0 (we took $\mathrm{\;d}X= 0.1$). Finally, $\mathrm{E}\left( e^{\beta X(t)} \,|\,T \ge t, X(s)=0 \right)$ for fixed $t>s$ was approximated by considering the further subset of processes for which $T \ge t$ and calculating the average value of $e^{\beta X(t)}$ within those subsets. The procedure of taking appropriate subsets (from the same database) was repeated for $X(s) = \pm 0.5$. These latter approximations are shown as the logarithm in solid (wiggly, because of Monte Carlo error) lines, the approximations in (12) (also logarithm) as dotted lines. Figures 6b–d show results when the reference situation is changed by choosing different values of $\theta$ (b), $\rho$ (c) and $\sigma ^2_\mathrm{tot}$ (d).

All curves in Fig. 6a–d start at $\beta X(s) = 0.25, 0, -0.25$, for $X(s) = 0.5, 0, -0.5$, respectively, at $t=s$. The parameters $\theta$ and $\rho$ determine the speed of attenuation for $t > s$ and the asymptotes for $t \rightarrow \infty$ of the regression coefficients $\beta _{\mathrm{LM}}(t \,|\,s)$ in the landmark analyses. Lower values of $\theta$ and $\rho$ imply a higher degree of attenuation. The second term $\frac{1}{2} \beta ^2 \sigma ^2_{\mathrm{tot}} (1 - \rho ^2(s,t))$ increases from 0 at $t=s$ to $\frac{1}{2} \beta ^2 \sigma ^2_{\mathrm{tot}}$ at $t \rightarrow \infty$ and is incorporated equally in each of the dotted curves with each plot, since it does not depend on *X*(*s*). Changing the value of $\sigma ^2_{\mathrm{tot}}$ [comparing (a) and (d)] only leads to vertical shifts of the dotted curves and does not change the relative distance for different values of *X*(*s*). Finally, the difference between the theoretical approximations shown in the dotted lines and the Monte Carlo approximations in the solid lines reflect the effect of selective removal (because of death), which was ignored in (8) and onwards. Compared to a situation with no removal of subjects because of death, for a given *X*(*s*), subjects with higher *X*(*t*) have a higher probability of being removed. As a result, subjects with lower *X*(*t*) remain in the population, which results in the dotted curves being pulled downwards. The total variance $\sigma ^2_{\mathrm{tot}}$, which was seen not to influence the theoretical $\beta _{\mathrm{LM}}(t \,|\,s)$ in (13), does influence this selective removal. This behaviour is quite similar to the selective removal of subjects with high frailty values in frailty models, the effect of which is also stronger with increasing frailty variance. Further simulation studies (not shown here) indicated that the effect of selective removal is stronger (i.e. the approximation of (12) less accurate) when the baseline death rate is increased, a phenomenon that is also present in frailty models.

## Data Illustration

We further illustrate our results using the well-known Stanford heart transplant data [6], consisting of 103 patients admitted to a waiting list for a heart transplant. The event time is time from admittance to the waiting list until death; interest is in the effect of a heart transplant on survival. Of the 103 patients, 69 received a heart transplant, and a total of 75 patients died, 45 with a heart transplant and 30 without a heart transplant. Median follow-up calculated by reverse Kaplan–Meier was 2.51 years. An important covariate predictive of the effect of heart transplant is the mismatch score, measured for those patients with a heart transplant. It is a continuous score derived from antibody responses of pregnant women [6] and reflects the degree of incompatibility (based on tissue typing) between the donor and recipient. Median mismatch score was 1.08, with 0.75 and 1.58 as 25th and 75th percentiles. Because different effects of the heart transplant may be expected for patients with a high mismatch score and patients with a low mismatch score, we distinguish between patients with a mismatch score in the highest quartile and the rest. Four patients with a heart transplant and no mismatch score (because no tissue typing was performed) are included in the larger set of patients with a mismatch score $\le 1.58$. We define two time-dependent covariates of the type studied in Sect. 2.1: $X_1(t) = 1$, if the patient has received a heart transplant before time *t* with a mismatch score higher than 1.58 and 0 otherwise, and $X_2(t) = 1$, if the patient has received a heart transplant before time *t* with a mismatch score less than or equal to 1.58 and 0 otherwise. A time-dependent Cox regression with $X_1(t)$ and $X_2(t)$ as time-dependent covariates resulted in estimated regression coefficients of 0.605 with a standard error (SE) of 0.386 (hazard ratio; 95 % confidence interval 1.83; 0.86–3.90) for $X_1(t)$, and −0.031 with a standard error (SE) of 0.319 (hazard ratio; 95 % confidence interval 0.97; 0.52–1.81) for $X_2(t)$. The tentative conclusion, which has to be seen in the light of the fact that heart transplantation was in its infancy during data collection, seems to be that heart transplants with a high mismatch score do more harm than good and that no clear effect can be seen of heart transplants with a low/medium mismatch score. Since differences between time-dependent Cox regression and landmarking can be most clearly seen for time-dependent covariates with large effects, we will focus on the effect of $X_1(t)$ in a number of landmark models.

Most of the heart transplants in the data occur in the first couple of months, so we take $s\,$=$\,1, 1.5, 2$ months as landmark time points for illustration. Earlier and later landmark time points result in numbers of transplant with (mismatch) score > 1.58 of less than ten. Table 1 gathers the results of the time-dependent Cox regression and of the three landmark analyses.

**Table 1:** Results of time-dependent Cox regression and landmark analyses at landmark time points $s=1, 1.5, 2$ months

*s*	*n*	Transplants	Death	$\beta$(SE)	HR (95 % CI)
		(score $$>1.58$$)			
Time-dependent Cox	103	16 (15.6 %)	75 (72.8 %)	0.605 (0.386)	1.83 (0.86–3.90)
1 month	74	10 (13.5 %)	48 (64.9 %)	0.560 (0.444)	1.75 (0.73–4.18)
1.5 months	65	10 (15.4 %)	40 (61.5 %)	0.255 (0.492)	1.29 (0.49–3.39)
2 months	57	10 (17.5 %)	32 (56.1 %)	0.431 (0.533)	1.54 (0.54–4.37)

It is clear that, compared to the hazard ratio of 1.83 of the time-dependent Cox regression, the estimated hazard ratios are indeed attenuated towards 1, as predicted by our results. The degree of attenuation is determined by many factors: $\pi _{01}(s,t)$ in Eq. (4), possible time-varying effects $\beta (t)$ in the time-dependent Cox model, and by the degree of censoring [see Eq. (6)], which makes it hard to compare the results of the different landmark models.

## Discussion

In this paper we derived relations between the regression coefficients obtained in a landmark analysis and those of a time-dependent Cox regression, when interest is in the effect of a time-dependent covariate on survival. In case the time-dependent covariate has no effect on survival at all, i.e. when the time-dependent Cox regression coefficient is identically 0, the landmark regression coefficient is identically 0 as well. Otherwise the time-dependent Cox regression coefficient will be attenuated. Different formulas apply for dichotomous and continuous covariates, but the degree of attenuation is mainly determined by how quickly the value of the time-dependent covariate changes over time. For dichotomous time-dependent covariates this is expressed in the prevalence probabilities, while for the Ornstein–Uhlenbeck example it is expressed in terms of the intraclass correlation and the $\theta$ parameter describing the degree of mean reversal.

We did not study the effects of measurement error (misclassification error in the case of dichotomous time-dependent covariates) or ageing due to infrequent measurements. The approximations in Sect. 2.2.1 can be adapted to account for that, in the spirit of [2], and they will result in a further attenuation of the regression coefficient of the landmark analysis. The main reason for not considering this aspect in detail is that landmark analysis and time-dependent Cox regression analysis will both be affected by these complications.
